# Nano-Bio-Genesis: tracing the rise of nanotechnology and nanobiotechnology as 'big science'

**DOI:** 10.1186/1747-5333-2-3

**Published:** 2007-07-14

**Authors:** Rajan P Kulkarni

**Affiliations:** 1 Centre for Economics & Policy, Institute for Manufacturing, Department of Engineering Cambridge University, Cambridge CB2 1RX, UK

## Abstract

Nanotechnology research has lately been of intense interest because of its perceived potential for many diverse fields of science. Nanotechnology's tools have found application in diverse fields, from biology to device physics. By the 1990s, there was a concerted effort in the United States to develop a national initiative to promote such research. The success of this effort led to a significant influx of resources and interest in nanotechnology and nanobiotechnology and to the establishment of centralized research programs and facilities. Further government initiatives (at federal, state, and local levels) have firmly cemented these disciplines as 'big science,' with efforts increasingly concentrated at select laboratories and centers. In many respects, these trends mirror certain changes in academic science over the past twenty years, with a greater emphasis on applied science and research that can be more directly utilized for commercial applications.

We also compare the National Nanotechnology Initiative and its successors to the Human Genome Project, another large-scale, government funded initiative. These precedents made acceptance of shifts in nanotechnology easier for researchers to accept, as they followed trends already established within most fields of science. Finally, these trends are examined in the design of technologies for detection and treatment of cancer, through the Alliance for Nanotechnology in Cancer initiative of the National Cancer Institute. Federal funding of these nanotechnology initiatives has allowed for expansion into diverse fields and the impetus for expanding the scope of research of several fields, especially biomedicine, though the ultimate utility and impact of all these efforts remains to be seen.

## Background

On September 13, 2004, at a conference room at the National Institutes of Health (NIH) in Bethesda, the National Cancer Institute (NCI) formally launched the Alliance for Nanotechnology in Cancer to an audience of reporters, government officials, and scientists. The program commenced with general speeches detailing the initiative, exploring safety and ethical issues, and explaining the potential of nanotechnology for cancer applications for the benefit of the non-technical members of the audience. Later, a scientific round-table followed, featuring prominent cancer and nanotechnology researchers including Richard Smalley (inventor of C_60 _and winner of the Nobel Prize), Mauro Ferrari of Ohio State University, and Andrew von Eschenbach, director of the NCI. During the roundtable, the speakers discussed various options for researchers to join the efforts of the Alliance [Note A]. This program, initially scheduled to last for five years with $144 million in funding, was designed to develop applications of nanotechnology for cancer diagnosis and treatment, yet it only represented a fraction of total spending on nanobiotechnology applications. The subsequent grant applications to these NCI programs signaled the increasing interest of the biological and physical science communities in utilizing nanotechnology to address issues of cancer biology, a sizable expansion of the burgeoning nanobiotechnology movement thanks to federal patronage.

To trace the genesis of this alliance, including the science and politics fundamentally underlying the program, it is necessary to briefly visit the increase in multidisciplinary research between biology and other fields from the 1970s onwards [Note B]. The idea of directed manipulations at the nanoscale finally became reality by the last decades of the twentieth century. Research in biology demonstrated that the fundamental reactions of life also occur at the nanoscale. Though scientists did manage to combine these two seemingly disparate fields, this combination was by no means inevitable.

Though there had been increased linkage between academia and industry for physics and materials science research since World War II, and a greater tendency towards more collaborative and centralized projects, biological research was relatively isolated from these trends until more recently. The Manhattan Project augmented the evolution towards 'big science' for academic physics researchers and led to increasing collaboration and contacts with industrial research (as many government sponsored contracts were awarded to industry as well); however, it was not until the Human Genome Project and the Bayh-Dole Act of the 1980s and 1990s that similar trends were seen in the biological sciences. Academic nanotechnology research has also followed a similar path, with large collaborative projects (both among universities and in partnership with industry) accelerated after the injection of hundreds of millions of federal research dollars. Nanotechnology, with its emphasis on device development, held much commercial potential and thus became viewed as a perfect opportunity for government sponsorship.

In the United States, a dedicated group of scientific officials among various federal agencies led the charge to develop a coherent, unified nanotechnology initiative. America's response to the growing global challenge was this program, which later became the National Nanotechnology Initiative (NNI). Once the NNI commenced, it gained a momentum of its own, and subsequent developments, including the 21^st ^century Nanotechnology Research and Development Act (NRDA), were much easier to justify especially with the notion that American scientific superiority was at stake, indelibly entrenching nanotech as 'big science.'

Initially an afterthought, biological applications of nanotechnology (nanobiotechnology) soon became focus of the federally sponsored research programs. Iijima's successful fabrication of carbon nanotubes in 1991 demonstrated the feasibility of bottom-up nanosynthesis, which involved the creation of larger structures by directed self-assembly from individual components. Shortly thereafter, scientists began to explore the viability of experimentally interfacing biology with nanotechnology, including the assembly of DNA sequences into three-dimension structures and the attempt to create biological transistors [1,2]. These projects highlighted the potential for creating novel devices, though fully functional applications would require significantly more research.

The NNI and the NRDA afterwards prominently featured biological applications as areas of high priority. With the rise of the NIH as the pre-eminent federal funding agency (with almost eight times as much money as the next nearest agency), there were billions of dollars potentially available for nano research specifically directed towards the life sciences. One of the first NIH-related agencies to devise such a nano-biological framework was the NCI. Cancer has been a specially designated research topic for over thirty years, beginning with President Richard Nixon's War on Cancer in the early 1970s. The NCI's Cancer Nanotechnology Plan (CNPlan) represented a direct link to this focus on cancer and the desire to focus the tools of nanotechnology towards problems of cancer detection and treatment. Given the trends mentioned, this combination was perhaps inevitable, but will such research ultimately be useful to biologists and clinicians? Despite the rosy predictions of certain researchers, it is too soon to predict the outcome.

This article focuses on the rise of nanotechnology and nanobiotechnology under the patronage of various sponsors (both government and private foundations). We place special emphasis on the confluence of nanoscience with biological research and how the influence of such sponsorship affected this research. We further detail the similarity of many of the current nano initiatives to past large-scale government sponsored projects, specifically the Human Genome Project. Finally, the trends influencing the CNPlan are explored. While the ultimate impact of nanotechnology on diverse fields is still yet to be determined, the progression of developments has already had significant impact on the topics of research as well as on federal research disbursements and helped to promote these fields as big science.

## Results and Discussion

### Nano Meets Bio

To understand the confluence between biology and nanoscience, it is necessary to return to the seminal events of molecular biology in the 1950s. James Watson and Francis Crick's discovery of the structure of the DNA molecule in 1953 opened a powerful new avenue of research. Subsequent research enabled the elucidation of the genetic code and the mechanisms of transcription and translation, the processes by which information encoded in DNA is translated into RNA and ultimately into proteins (the so-called 'Central Dogma' of biology) [3]. Research over the next half century helped to build our knowledge of the fundamental processes of enzymes and cells and the molecular basis for many of the reactions of living organisms.

Though most biological research deals with processes and events that occur on the nanoscale, this research was not considered part of nanotechnology, as such molecules could not be directly manipulated. The linkage between the two was tenuously formed, building in incremental steps and with many experiments in between. By the early 1970s, biologists had learned much about how biomolecules functioned within the cellular environment; they soon realized that they could engineer DNA and protein sequences at will using many of the enzymes that the cell normally used for life processes. Recombinant DNA technologies were enabled with the isolation of restriction enzymes (the first by Dan Nathans) to cut DNA pieces and ligases to attach the DNA fragments back together. The discovery of polymerase chain reaction (PCR) and the development of high throughput DNA synthesis and sequencing technologies were additional important milestones [4]. Scientists could now amplify virtually any DNA sequence and begin to undertake genome sequencing projects thanks to increasingly affordable DNA sequencers. These technologies greatly expanded the scope and scale of biological experimentation and innovation.

By the 1990s, scientists began to design experiments to specifically couple biology with nanofabricated devices and tools [5-7]. Though the length scales were compatible, there were significant challenges involved. Biological systems are fundamentally wet and organic, whereas most nanofabricated systems are hydrophobic and made of inorganic materials (usually silicon-based). Though ideas of nanobiotechnology had circulated among the scientific community and general public for many years, actual progress in this field only began when the initial seminal advances of each contributory field (biotechnology and nanotechnology) had come to fruition by the early 1990s. These developments attracted many scientists interested in the interface between the two. However, one major problem was how to physically couple the two divergent systems [8]. Some scientists circumvented this problem by creating nanomachines made solely of natural molecules; two notable examples were Nadrian Seeman's complex 3-D structures created solely of DNA and Leonard Adleman's utilization of DNA to perform computation [9,10]. Others discovered or developed new coupling chemistries in order to covalently bond organic and inorganic substrates. The collaboration of scientists from both fields has been important for refining tools used for nanobiotechnology and in building the path towards functional hybrid devices. For example, Carlo Montemagno of UCLA recently created hybrid nanomachines composed of an inorganic nanopropeller with a biomolecular motor that could use adenosine triphosphate (ATP) for energy [11]. Other device researchers have created more complex hybrid functional micro-electro mechanical systems (MEMS) and even nano-electro mechanical systems (NEMS) devices composed of a combination of synthetic and biological components [12].

Even with the increasing public awareness of nanotechnology, and with scientists initiating more projects, the federal government's response in the late 1980s and early 1990s was rather sparse. Academic scientists hoping to receive funding had to apply through existing programs offered by the NSF or other granting agencies; there was no specific focus on nanotechnology. However, the early 1990s marked a particularly fortuitous moment for nanotechnology, as the Cold War had ended and America was concerned about its scientific superiority and was looking for promising areas of research that could help maintain its global scientific and economic edge. Thanks to this attention, and the involvement of government officials such as Mihail Roco at NSF, the time was ripe by the 1990s for an organized government project. This effort culminated in the National Nanotechnology Initiative (NNI) and its progeny and sent nanotechnology on the path towards 'big science,' opening the door for large-scale nanobiotechnology projects [13].

### Big Science Requires Big Money

With great fanfare, President William J. Clinton announced the National Nanotechnology Initiative (NNI) to a packed audience at Caltech on January 21, 2000 [14]. Through this initiative, President Clinton more than doubled the federal funding for nanotechnology research to almost $500 million for fiscal year (FY) 2001; since then, the budget has doubled once again to over $1 billion for FY 2006 [15]. Both commercial and state/regional investments in nanotechnology similarly increased significantly during this period. As Patrick McCray has correctly argued, the NNI emerged at the moment when politicians and policy makers were working to respond to growing international competition in the economic and technological spheres [16]. Over the last decade, worldwide governmental, academic, and corporate support for nanotechnology research has increased nearly exponentially, with new corporate and academic projects and initiatives announced monthly. As a result of this support, the transformation of nanotechnology into a component of 'big science' is nearly complete.

This paradigm shift towards big science occurred in part due to the increasingly specialized and expensive tools required for cutting-edge nanoscale research. However, much of this change came as research funding focused on the establishment of federally supported nanotechnology centers (designated by the NNI) at various universities and research laboratories [Note C]. For the fortunate locations, being a 'chosen site' signified access to millions of dollars of research funding. Other researchers would have to compete for the remaining dollars.

Before enactment of the NNI and related initiatives, federal support for nano research was sporadic at best; there was no distinct mechanism for obtaining financial support [16]. As a result, research proceeded through the early 1990s at various academic centers with intermittent grant support from federal agencies such as NSF or the Department of Energy (DOE) [17]. With no promise of continuity, researchers were at the mercy of grant officers and review committees to maintain research support. Excitement at the prospects of nanotechnology drew more researchers into these fields, thus creating increasing pressure for a more specific and formalized federal program.

Efforts by Mihail Roco of the NSF and others culminated in the National Nanotechnology Initiative (NNI) [Note D]. The NNI emphasized eleven areas, the so-called 'Grand Challenges,' collaborative projects intended for interagency funding. The remainder of the money would be used to establish a national infrastructure with 'centers of excellence' where researchers could collaborate and access advanced equipment. Final approval of the NNI came in the fall of 2000, with an allocation of $465 million.

This emphasis on nanoscale research continued even with the change of presidential administrations in early 2001. President Bush proposed a modest increase in the budget for the NNI in the FY 2002 budget; Congress topped this by approving an additional 15% increase to a total of $600 million [18]. After the terrorist attacks of September 11, 2001, nanotechnology research was increasingly promoted as a means for detecting chemical and biological toxin precursors and for helping to increase national security. Because trace detection had been one of the original selling features of nanotechnology, this research received even greater funding [16]. Any lingering doubts of the efficacy of nano research had long since disappeared, and Congress was very receptive to calls for additional funding.

One limitation of the NNI was that it was a short-term initiative and therefore subject to the yearly funding allocation process. The National Research Council's (NRC) initial review of the NNI, entitled *Small Wonders, Endless Frontiers*, provided an impetus for formally codifying the nanotechnology push into law to ensure continuity of funding, which Congress soon addressed. In early 2003, Representative Sherwood Boehlert introduced the Nanotechnology Research and Development Act of 2003 to the House of Representatives [19], and Senator Ron Wyden introduced the 21^st ^Century Nanotechnology Research and Development Act (hereafter referred to as the NRDA) to the Senate floor [Note E]. The House was first to conduct hearings on the merits of this proposal, with the Science Committee holding hearings twice, on March 19 and April 9, 2003. By this time, nanotechnology had firm bipartisan support and Congress was generally pleased with the implementation of the NNI. Passage of the bill came swiftly on May 7, and the bill proceeded to the Senate for consideration [20].

The Senate Committee on Commerce, Science, and Transportation held hearings on May 1. At these hearings, E. Clayton Teague, the recently appointed full-time director of the National Nanotechnology Coordination Office (NNCO), testified in support of the legislation. In his oral and written testimony, he emphasized the potential of nanotechnology, especially for commercial applications and technology transfer, and urged support for continuing such research, mentioning that "...this great promise must be tempered with the realization that our nanotechnology capabilities are now at an embryonic stage. It has taken us twenty years to progress from the ability to see atoms, then to manipulate them, and finally a few years ago to build a simple three atom structure – twenty years [21]." He highlighted the progress made through the NNI and the efforts of the relevant federal agencies to promote technology transfer and commercialization applications while emphasizing the applied nature of their research [22].

The National Institutes of Health also submitted written testimony in support, stressing the potential of biomedical applications and mentioning that nanotechnology "has the potential to radically change the study of basic biological mechanisms as well as to significantly improve ... treatment of diseases and adverse medical conditions [23]." They further stressed the interdisciplinary nature this work and the need to support research among various fields, including engineering, physical science, biology, and medicine. The benefits were stated in no uncertain terms – nanotechnology could be harnessed to cure disease and improve the human condition, with the ultimate goal of a world where "diseases are diagnosed and prevented or treated at early stages [23]." Implicit was the promise that this was possible if Congress would allocate the necessary funds.

While passage of the NNI had required its backers to clear large hurdles, there was little uncertainty surrounding the fate of this bill, as the senators were in favor of increased support for nanotechnology. Once the NNI received funding, there was less incentive to backtrack, especially given the glowing testimony of the witnesses and the strong support of the scientific and business communities. By mid-September, the Senate committee had referred the bill to the full Senate [Note F], and final passage came on November 19 by unanimous consent. Less than three weeks later, on December 3, 2003, the 21^st ^century Nanotechnology Research and Development Act became law [24].

The NRDA authorized the president to implement a national nanotechnology program administered by the National Science and Technology Council (NSTC). Much as the NNI before, this program was intended to emphasize interdisciplinary work and to "accelerat[e] the deployment and application of nanotechnology research and development in the private sector [24]." Congress specifically allocated funding for additional centralized nanotechnology research centers and directed the president to establish a National Nanotechnology Coordination Office to provide technical support for implementation of the NRDA and to conduct public outreach efforts. The act also required President Bush to designate a National Nanotechnology Advisory Panel (NNAP) to provide advice about nanotechnology trends and the efficacy of implementation of the NRDA [24]. Overall, nanotechnology funding levels reached $3.7 billion for the subsequent four years, representing a significant increase from the previous funding levels established under the NNI.

In a recent strategic update to the NNI, mandated by the NRDA and issued in December 2004, the Nanoscale Science, Engineering, and Technology Subcommittee of the NSTC examined many of the initial issues of the NNI and also looked to its potential for the next ten years [25]. Overall, this report was supportive of the progress of NNI supported research and made confident predictions that the future would yield even better results and assist the United States in maintaining its premier economic and research advantages, stating, "...the NNI will provide a balanced and coordinated investment in the program component areas and in a broad spectrum of applications. This will ensure that the United States remains a global leader in the responsible development of nanotechnology and secures the resulting benefits to the economy, to national security, and to the quality of life of all citizens [25]."

This report identified four major goals of the NNI, which included maintaining a strong R&D program, facilitating technology transfer for economic benefit, developing infrastructure to advance nanotechnology, and supporting responsible development of nanotechnology. As mandated by the NRDA, the original 'grand challenges' of the NNI were replaced by seven 'program component areas [25].' The report also emphasized the development of research centers, including already existing centers and future planned projects. Investment in infrastructure remains the major component of government nanotechnology spending. In total, the NSF, DOD, and NASA have established 24 research centers at both academic and government laboratories which provide advanced technologies and equipment for collaborative efforts [26]. These centers represent the 'favored' sites for government funding and have received a disproportionate share of funds, while other academic researchers have had to compete for the remainder. Despite this centralization of nanotechnology research, there has been little dissent due to the massive quantities of federal dollars invested since 2001 and additional monies offered by other concerns. Political support for nanotechnology is now a fait accompli, and thanks to this federal largesse, nanotechnology has firmly been established as big science.

### Regional and Private Nanotechnology Initiatives

Meanwhile, state and local governments had begun to enact supplementary initiatives to promote nanotechnology research and cement connections among industry, academia, and government. State governments especially saw nanotechnology as a means for boosting regional economies and luring companies into bringing prestigious and high-paying jobs to certain areas. In an attempt to attract corporations, many regions offered tax or other relocation incentives. A small number of states allocated supplemental funds to state-supported universities, directed specifically towards nanotechnology research. However, the scope and size of these initiatives varied widely, ranging from the California Nanosystems Institute (CNSI), designed to provide infrastructure funding at UCLA and UCSB to the level of $100 million for four years, to the South Carolina NanoCenter, funded with a one-time grant of $1 million [27]. Many localities were trying to join the nano-bandwagon in an attempt to emulate the success of past regional investments in biotechnology or information technology, especially those in California (Silicon Valley) and Massachusetts (Route 128).

Given the intense regional interest in nanotechnology research, the Department of Commerce and the NNCO convened a workshop in late 2003 to gather leaders involved in establishing state and regional nanotechnology initiatives in order to enhance communication among and across these different programs [27]. The focus of this meeting was to leverage the combined efforts of the federal government with state government efforts to prevent inefficiency and promote synergy if possible. As the program summary noted, not all of these initiatives would succeed; however, the report aimed to elucidate the characteristics of successful ventures. Each local initiative was encouraged to find a niche area to focus on for developing distinctive competencies to leverage partnerships with corporate efforts [27].

Perhaps the most successful example of such an initiative is the California Nanosystems Institute (CNSI), launched almost concomitantly with the federal NNI. The CNSI was proposed as one of a handful of science research institutes designated for the various campuses of the University of California system. Then-governor Gray Davis formally introduced these research centers in his 2000–2001 budget, which were enacted in California Assembly Bill 2883 of 2000. The impetus for the establishment of these institutes, which came to be known as the California Institutes for Science and Innovation, was to "ensure that California maintains and expands its role at the leading edge of technological innovation in the 21st century" and to "give rise to world-class centers for strategic innovation that combine excellence in cutting-edge research with collaboration and training for our next generation of scientists and technological leaders [28]." They included the California Institute for Quantitative Biological Research (QB3), the CNSI at UCSB and UCLA, and the California Institute for Telecommunications and Information Technology (Cal-IT2) [29] [Note G]. This act formally established these centers throughout the University of California system and allocated a total of $300 million over 4 years, with each receiving $25 million per year from the state. State funding was contingent on securing external 2:1 matching private and federal grants [30].

When the CNSI was established, lawmakers estimated that it would provide at least $350 million in nanotechnology funding over the four year period, second only to the NNI in financial commitment [31]. In particular, state funding of the CNSI provided for the physical establishment of buildings dedicated to nanoscience research groups and for housing relevant equipment in support of these endeavors at UCLA and UCSB. The state expected these universities to find sponsors to fund everyday operating expenses; this was not expected to be problematic, given significant corporate interest in supporting applied academic research. Indeed, shortly after Gray Davis' announcement, UCLA had already lined up over thirty partners for the CNSI [32]. The explicit goals of the CNSI were to leverage California's investment with federal and private funds in order to create a nanotechnology hub where such research could flourish. The new CNSI buildings would serve as central locations for equipment and researchers with the goal of fostering collaboration and interdisciplinary thinking among the scientists.

Within the first five years of operations, the CNSI attracted the external financial support mandated by the legislature and began an ambitious expansion program to establish additional laboratories at UCSB and UCLA. With this level of support, the NSF designated it as a NanoScience and Engineering Center (NSEC) and awarded an $18 million grant to create a research and fabrication center for nanomanufacturing, entitled the Center for Scalable and Integrated Nanomanufacturing [33]. The 2005 annual report from the UCLA branch of CNSI markedly emphasized collaborative efforts, both between academic institutions and with corporate interests [34]. The report constantly stressed the benefits of state investment in CNSI, to remind the public that the allocation of tax dollars to this project would continue to produce tangible benefits. Fraser Stoddart, the CNSI director mentioned in his introduction, "With the appropriate investment, it can help create jobs, foster economic growth, and promote technological advancements that will improve the quality of life for the people in California and beyond [34]." In this era of limited government revenues, every dollar spent must be shown to generate economic benefit.

California's level of commitment and involvement soon became a model for other states to follow, though none could provide as much financial support. Massachusetts, home to many distinguished academic institutions, looked to this example when identifying opportunities to leverage its academic and commercial strengths to promote nanotechnology. A report prepared for the state economic department mentioned these strengths and the history of technology transfer to startup and other commercial enterprises [35]. Given the strengths of their universities, especially MIT and Harvard, the authors expected that success would be forthcoming. They encouraged a more conducive environment for scientific endeavors and technology transfer, with less state regulation. However, funds for such research were not readily forthcoming; instead, the report mentioned that federal dollars should be leveraged for maximal benefit. Other states echoed many of these issues as they looked to join the nanotechnology bandwagon to capture some of the potential economic benefits that such research promised.

Meanwhile, individual private benefactors and foundations entered the research field through directed grants or the establishment of research centers, usually at universities. One heavily publicized effort was spearheaded by Fred Kavli; through the Kavli Foundation, he began to establish scientific research institutes, specifically focused on nanoscience, neuroscience, and astrophysics, at various universities throughout the world [36]. Kavli, a Norwegian entrepreneur and founder of Kavlico, a firm that manufactured sensors for aircraft and automobiles, used the proceeds from the sale of his company in 2000 to establish the Kavli Foundation to support basic science research at universities worldwide. His first gift was a donation of $7.5 million to UCSB in 2001 to establish the Kavli Institute for Theoretical Physics. In the following three years, Kavli established nine more institutes [37]. Though the interest from each gift is rather small in comparison to the endowment of these universities or the amount of government grants that each receives, the money is unrestricted and allows great freedom in choosing research directions. One benefit of gifts from small foundations (assets < $100 million) is that they allow scientists to respond rapidly to emerging research needs. Kavli hoped to use his gifts as 'seed money' to help scientists explore promising but unproven research areas, projects that would typically have trouble receiving government funding. He noted, "I'm looking for highly leveraged situations where institutions are putting in a large share [of their own funds] [38]." In the era of constrained federal budgets, this flexibility would be increasingly important for exploring novel areas of research.

Three of the Kavli institutes were focused on nanoscience and nanotechnology research: those at Cornell, the Delft Institute of Technology in the Netherlands, and at Caltech. Caltech established the Kavli Nanoscience Institute (KNI) in 2004 with an initial $7.5 million gift from the Kavli Foundation. Along with support from the Moore Foundation, the outlines of a broad nanotechnology institute were created, with specific foci on nanobiotechnology and nanophotonics [39]. Michael Roukes, Caltech professor of physics, was named as director, and he mentioned that "the primary emphases of the KNI will be on nanobiotechnology, which merges nanodevice engineering with the molecular and cellular machinery of living systems, and nanophotonics, which employs new materials technology and nanofabrication processes to develop novel devices such as optically active waveguides and microlasers. Central to both of these endeavors is large-scale integration of nanosystems [40]." Caltech's KNI was formally dedicated at the inaugural Kavli Nanoscience symposium on October 24, 2005 [41].

In an attempt to increase public interest and consciousness of Kavli's chosen fields, he established the Kavli Prizes in Nanoscience, Neuroscience, and Astrophysics in 2005. To be awarded every two years by the Norwegian Academy of Sciences, with a value of $1 million each, these prizes are intended to be complementary to the most prestigious awards in science (e.g. Nobel, Crafoord, Gruber). Kavli's motivation was to "raise the public awareness of science," since societal support would be critical "in order for the politicians to support spending the funds," as most research funding for these fields came from national governments [42]. Just as the Nobel prizes beforehand, such awards are likely to attract media attention and thus the public's awareness to significant advances in these fields.

### Centralization of Nano Research

One major consequence of the NNI and other nanotechnology initiatives has been the establishment of this discipline as 'big science,' with centralization of significant research dollars and resources at select institutions and the appointment of certain laboratories to receive support for large-scale, integrated nanotechnology projects. In the state of California alone, there were two major grants to establish nanoscience institutes at three leading universities (two state funded and one private). While researchers at other institutions have pursued nanoscience research, they have had to make do with limited government funds from agencies such as NSF or NIH, without the infrastructure support that the nanoscience institutes can offer. Furthermore, grant programs have begun to emphasize cross-disciplinary projects that require input from physicists, biologists, and other scientists, both intramurally and between institutions. It is apparent that in this third 'mega-era' of science policy [Note H], research resources are becoming even more centralized with the increasing need for collaborative and multidisciplinary projects in order to satisfy the mandate (both from politicians and the public) for research that is directed towards specific economic and societal needs.

Federal grant agencies have now dedicated a significant portion of grants towards interdisciplinary work and away from the traditional fields of chemistry, biology, and physics. As described, the NNI has supported the establishment of nanotechnology centers and user facilities at national research laboratories and universities across the country, with the goal of establishing infrastructure due to the high costs of equipment [43]. These resources are usually concentrated at the top-tier research universities because these institutions have the most prolific researchers and large research budgets to fund additional work.

In many ways, the NNI is accelerating trends seen at academic research institutions in recent years. According to Roger Geiger, the first formal links between academia and industry came in the 1920s (notable examples being MIT and Michigan's Engineering Department); however, the significant majority of links were forged in the 1980s [44]. As a result of these increased contacts, Daniel Kleinman argues that there has been a convergence (albeit asymmetric) in research practice between industry and university, concomitant with increasing emphasis on university-industry relations. Such relations are now explicitly encouraged by government and have become more formalized and extensive [45].

Similar trends are evident in the centralization of research facilities and projects. The idea of centralized research laboratories for physics and engineering was significantly strengthened during World War II with the need to concentrate efforts on developing the nuclear bomb and other projects of military interest. Such organized research units were often under or later transferred to university control. However, there was little pressure towards such facilities in other disciplines until very recently. This trend towards centralized research facilities and cooperative projects (what John Ziman terms 'collectivization') is driven partly by researchers' need for increasingly expensive and intricate instruments [45]. Because modern research is increasingly transdisciplinary and requires a great deal of specialization, individuals working in isolation cannot generally solve major scientific problems any longer.

Another hallmark is the constraint on funding and the limits to its growth. Historically, science could easily grow exponentially as very few individuals were engaged in research. However, with federal science budgets of developed nations at 2–3% of gross domestic product, there is very little room for augmented funding. As a result, increased allocations for nanotechnology mean less money for other disciplines. Science is under significant pressure to provide greater societal benefits for the money allocated. Given societal and epistemic changes, scientists have begun to internalize these trends and emphasize the direct and indirect benefits of their work in response, especially short-term utility and applicability. Funding agencies are increasingly requiring that grant applicants stress the practical benefits of their research, and most academic scientists have little choice but to comply. Such policy shifts have been a direct result of the increasingly competitive environment of the past thirty years.

Government scientific priorities underwent a marked shift in the late 1970s and early 1980s due to global economic considerations. The old paradigm that funding pure basic science was useful because such research would provide the knowledge for new technologies was challenged. A poignant criticism was that this work was ultimately irrelevant for the economy and that America did not have the luxury of funding this while other nations were supporting applied and industrial research. Increasingly, corporate leaders and politicians adopted the view that the government should encourage 'productive' research, work that would lead to practical applications and boost the economy [46]. One result was a shift in research priorities at the end of the Cold War away from pure basic science (such as particle or high-energy physics) towards more applied or practical basic sciences (e.g., the biological sciences, as such research was often relevant for pharmaceutical or biotechnology products), as reflected in the budgets of the relevant granting agencies. Throughout the 1990s, the budgets for NSF and NASA remained stagnant while the budget for the NIH increased fivefold [Note I]. By the time Congress debated the NNI, there was no question that the initiative would focus on applied technologies.

In the initial NNI proposal, the greatest stress was on projects that could be completed within a 5 year time window, to emphasize its focus on applications that would yield commercially relevant results in the shortest time [43]. The President's Council of Advisors on Science and Technology (PCAST) report in support of the NNI had to justify the need for funding longer term research by noting industry's aversion to funding such endeavors, while still maintaining that the research was viable and only needed continuous government support [47]. Despite this apparent support for longer term work, most of the initial awards were for evolutionary research that had the best prospects for near-term success. In response, the NAS encouraged the NNI to support higher-risk, exploratory research that had greater potential for significant breakthroughs [48]. However, mindful of the economic emphasis of the NNI, the NAS also recommended promoting research transfer to society and fostering industrial partnerships to accelerate commercialization: "In developing nanoscale science and technology as a competence of the national scientific and industrial establishment, the federal government must promote, cooperate, seed, and leverage [48]."

This transformation of government research priorities to an applied science focus was well underway by the time the NNI Strategic Plan Update was published in December 2004. The update explicitly stated the fiscal rationale for nanotechnology funding, namely increased economic development and prosperity: "Towards this vision, the NNI will expedite the discovery, development, and deployment of nanotechnology in order to achieve responsible and sustainable economic benefits...Nanotechnology R&D has led to substantial increases in scientific knowledge, publications, patents, and new jobs and businesses in this area. Much of this success is directly or indirectly based on the results of Federally funded R&D [43]." Universities were especially encouraged to collaborate amongst themselves and with industry to realize the commercial and societal potential of this research.

### The NNI and the Academic-Industrial Complex

Given its emphasis on applied research and the explicit bias in support of projects of commercial interest, the NNI has significantly promoted the continued blurring of any distinction between academic and industrial research and explicitly encouraged stronger links between the two. This trend has been evident for many years in engineering disciplines, as university-industry relations have been strengthening for many years in these areas (as Roger Geiger has noted), a result of the 'practical' nature of most engineering research, whereas basic science had been more insulated [44,49]. As Daniel Kleinman mentioned, academic science has begun to resemble industrial science with its focus on specific topics with defined goals (often for business applications) [50,51]. If scientific research is primarily intended for national economic benefit, then this trend is unavoidable. Many of the federal government's policies of the past twenty-five years have explicitly promoted this development by increasing the economic incentive to pursue research with distinct commercial and patent possibilities, as Ann Johnson has detailed [52].

With federal research budgets static or shrinking as a percentage of GDP, universities began to seek outside sources of funding [53]. There were a few private foundations that would disburse grants; however, these funds were usually intended for specific, targeted purposes. As a result, universities increasingly turned to industry for a steady source of money. In return for receiving unrestricted dollars which they could allocate as needed, universities and academic departments often agreed to exclusively license any resulting patents to the sponsoring companies. A well-publicized example was the agreement between the Department of Plant and Microbial Biology at University of California, Berkeley and Novartis to negotiate licenses for about 30% of all patents from the department on a first refusal basis in return for $25 million in support over five years beginning in 1998 [54-56]. Participating researchers agreed to submit their manuscripts to Novartis in advance of publication and to sign confidentiality agreements if they utilized the company's databases. Furthermore, Novartis received seats on the department's research advisory committees [57]. Although some academics protested against such commercialization because of the different cultures of academic versus industrial research, most acquiesced in this shift. Collaborations between academic labs and industry, once largely found among the engineering and applied sciences, were becoming increasingly common among all science departments as a means of garnering research funds.

The government already had mechanisms for directly supporting small business' research through Small Business Innovation Research (SBIR) and Small Business Technology Transfer (STTR) grants administered by the Small Business Administration (SBA); STTR grants specifically required businesses to collaborate with non-profit laboratory partners to qualify for funds [58]. To encourage this, Congress required that federal agencies receiving research funding of more than $100 million per year set aside 2.5% of these funds for small companies to conduct innovative research. Each program encompassed two phases of funding; phase I funding was to establish feasibility and businesses could receive up to $100,000 for one year. Phase II funding provided limited support for further research and development and for exploration of commercial opportunities; grants could range up to $750,000 over two years. Afterwards, the SBA expected the company to raise private funds to fully migrate the product into the competitive marketplace.

The negative aspects of the increasing commercialization of academic research include loss of control and the concomitant trend towards selective revelation of results. Industrial research is often based on the premise of trade secrets or proprietary information and companies are increasingly requiring academics who accept funding to keep research results private or at least delay publication until patents can be secured. Similarly, the previous openness of academic science is increasingly curtailed by the commercialization of research as academics more frequently seek patent protection for their own discoveries. In the extreme case, corporate interests can demand that academics seek prior permission before publishing, allowing for review of the data to potentially suppress findings that might negatively affect the company. While many universities seek clauses allowing unfettered publication, this is not always true. The case of Betty Dong, a pharmacy professor at University of California, San Francisco, illustrated the potential pitfalls of such corporate support. Dong had received a $250,000 grant from the Boots drug company to carry out a comparative study of Synthroid, a drug used to treat hypothyroidism, and three similar generic drugs [Note J]. The Boots Company wanted to demonstrate that the brand name drug was superior to the generic drugs. Dong found that the generic drugs were equally effective as Synthroid, and that the generics were significantly cheaper and would save consumers millions of dollars a year. Boots was displeased and managed to block publication of this data since Dong had signed a contract with the company that allowed them to control publication and UCSF was unwilling to defend her [59]. However, the resulting uproar and controversy within the scientific community forced Boots to relent and allow publication [Note K]. In many cases, the combination can be synergistic; in others, there can be mutual antagonism as the two cultures clash.

Increasingly, professors were founding startup companies based on research from their laboratories, often funded by government research grants. Thanks to the Bayh-Dole Act of 1980, which allowed universities to directly patent federally sponsored research without having to pay royalties or specifically negotiate licenses with the government [60], their discoveries were more easily patented and licensed, with royalties going to the inventors and their institutions. Universities, interested in the bottom line, were happy to acquiesce for a share of the profits [Note L]. By the late 1990s, there were few universities that did not aggressively pursue such opportunities and market their research to commercial interests. With stagnant federal research budgets and increasing bureaucracies, funds had to come from somewhere, and industry seemed an appropriate partner.

### Nanotechnology as 'Big Science': A Comparison to the Human Genome Project

The establishment of nanotechnology and nanobiotechnology as 'big science' followed centralizing trends in most scientific disciplines over the past sixty years as described. This development was perhaps less noticed given the establishment of large-scale, integrated projects and facilities in all major disciplines, including biology. Indeed, by the time the NNI was approved, the existence of large-scale, big science laboratories and projects was taken for granted in physics, engineering, and biology. The first major impetus came from the Manhattan Project, which established centralized laboratories for physics and certain engineering research, as described by Jeff Hughes [61]. However, biology did not experience such pressures until the establishment of the Human Genome Project (HGP), which have not been as extensively detailed. The progression of the HGP portended many of the policies and trends seen in the NNI, but with certain features unique to each. We identify certain forces and lessons from the HGP and discuss how they have affected the NNI and may impact further implementation.

In many significant aspects, the NNI is similar to the HGP. Both programs were initiated partly on the basis of bold claims made by scientists and other visionaries to advance their interests. However, unlike for nanotechnology, there were initially few companies performing genome sequencing research because of the prohibitive costs and the lack of immediate tangible benefits. The difficulties of organizing such a large scale project nonetheless remained. Robert Sinsheimer, a molecular biologist and chancellor of UC Santa Cruz, and Charles DeLisi, director of the Office of Health and Environment at the DOE spearheaded initial work on the HGP, just as Roco at the NSF provided a major impetus for the NNI [62].

Both initiatives were intended to be multidisciplinary efforts spanning different governmental institutes, and each was subjected to turf battles and infighting that limited the scope that could be accomplished and even threatened to derail portions at times. Beginning in 1985, DeLisi lobbied for money for the HGP and finally received $5.5 million for the DOE's genome effort in 1987 [62]. However, the DOE's push to begin genome sequencing bothered certain scientists at the NIH, as they worried that they would lose grant money as a result [63], while other scientists were worried of the loss of autonomy due to this large initiative. When Congress approved the final appropriations bill, both the NIH and the DOE received funding for genome research, with the NIH receiving 50 percent more than the DOE. The federal government had officially become inextricably involved in a large science project which eventually impacted all of molecular biology. In 1988, James Watson became head of the National Center for Human Genome Research, a newly created position within the NIH to manage this growing project [62]. The era of 'Big Biology' had arrived.

The tendency towards centralized biological research became especially prominent in the late 1980s with the establishment of NIH-funded genome centers. Though the NIH was the major government biomedical research laboratory, its budgets had trailed those of NSF or DOE. With the establishment of the HGP and related biomedical initiatives, the NIH received significant allocations from Congress to fund these centers at academic laboratories to serve as resources for the entire academic biology community. Thanks to this increased and continued support, the NIH has now become the largest federal granting agency, with a FY 2005 budget of over $20 billion.

Once the NNI had been proposed, it also faced similar critics who were worried that the creeping force of government support would turn nanotechnology into big science, just as genomics had converted certain aspects of biological research into big biology. It was difficult to see how the individual researcher could possibly innovate in such an environment. Indeed, implementation of the NNI and related nanotechnology funding by government has led to further expansion of big science through the establishment of user facilities and 'nanoscience institutes' at various universities and laboratories throughout the country. As a result of equipment requirements, funding has been concentrated at specific locations (the genome centers for the HGP, and the nanoscience centers for NNI) which obtained the bulk of federal spending for the respective projects.

The NNI received less criticism from scientists than the genome project, in part because many had become accustomed to big science through developments either in physics or biology. They realized that research budgets were unlikely to increase as in the past; it was therefore best to encourage funding increases for their areas of research or to spin their work to fall under the topic of interest. Even so, the NNI received exponentially more money in its first full year of funding compared to the HGP. One potential reason for this disparity is that the short-term prospects of nanotechnology appeared more lucrative than for the genome project, thanks to greater initial corporate interest in the NNI. Nonetheless, by its final years, the NIH provided more than $2 billion to the HGP and supported research at many genome centers throughout the country. Given the current rate of growth for nanotechnology research, it appears that the NNI will exceed this figure within the next five years.

One major difference between the two relates to the role of industry. The initial genome supporters urged funding because they argued that companies were unlikely to devote significant resources to sequencing genomes due to the immense cost and time (in the mid-1980s, sequencing was tedious and expensive and it was unknown how long sequencing even a fraction of the human genome would take). Furthermore, no one clearly understood the full significance of having these DNA sequences. On the other hand, the NNI has involved industry from the beginning and explicitly aimed to encourage research with business applications. With the NNI, small companies are specifically encouraged to apply for SBIR and STTR grants from the NIH and other federal grant agencies; such grants were unavailable or virtually unknown at the commencement of the HGP.

The trajectory of the HGP portended many of the developments and features of the NNI. Though the HGP was initially opposed by certain scientists worried about the consequences of big science, it ultimately gained widespread acceptance. In fact, the HGP began to develop its own inertia, gaining exponentially greater resources as the project continued and expanding into related research areas. A similar route can be seen for the NNI, with its initial growth occurring even faster. The relative abundance of resources for nanotechnology has attracted more research and provided an impetus for its expansion into other disciplines, especially for biological and medical applications, as these are seen as areas where nanotechnology might provide novel insights and potentially economically valuable returns.

### The Nano-War Against Cancer

One significant example of the expansion of nanobiotechnology initiatives has been towards applications for cancer research and treatments. A main selling point of the NNI was the potential of nanotechnology towards issues of human health and disease. Part of the interest in the linkage between nanobiotechnology and cancer is due to self-interest – research is more likely to be funded if targeted towards specific problems, especially health care. But certain researchers also believe that nanotechnology is a perfect fit because molecular biology deals with nano-sized molecules and processes [12]. A significant portion of this research has focused on the problem of identifying small quantities of molecules in solution, with the aim of detecting the presence of abnormal biological markers within serum to facilitate disease diagnosis and treatment [64].

Though nanotechnology has had limited impact on medical care and treatment to date, there is a sentiment among nanotechnology researchers that it will significantly impact the delivery and practice of medicine, though other scientists and physicians either are less convinced or are waiting to see the results [65,66]. Much of the initial optimism among nano-researchers comes from recent discoveries and inventions that interface nanotechnology with biological systems [12]. Cancer has been an especially important target because of the scope of the disease; in America alone, nearly 1.5 million individuals are diagnosed with cancer each year [67]. According to its promoters, the true promise of nanotechnology lies in its potential towards specific and individualized medicine based on improved detection and treatment modalities [68].

Certain doctors and cancer researchers see these treatments as the logical progression of cancer therapy [69]. According to Andrew von Eschenbach, former director of the NCI, "the future of oncology – and the opportunity to eliminate the suffering and death due to cancer – will hinge on our ability to confront cancer at its molecular level [67]." They see nanotechnology as a key component to facilitating this transition. Because of its scope, there is greater optimism that its tools can be brought to bear upon many of the issues facing oncology. However, others are more circumspect about the possibilities, acknowledging the potential for enhancing research and willing to utilize any useful discoveries, but cognizant of the potential difficulties of biological and cancer systems [66].

Although the claims from supporters has been reminiscent of initial talk about nanotechnology (especially from Eric Drexler), initial, short-term research work has been incremental and evolutionary in nature and has largely focused on developing novel and ultrasensitive diagnostic tools for the detection of cancer cells and markers secreted by these cells at very low quantities [70]. The goal is to identify cancerous or pre-cancerous cells before the tumor has a chance to grow and metastasize, as it is much easier to target the cancer with specific drugs at an early stage [12]. Other groups have worked on detecting tumors within animals, for identification for biopsy purposes and surgical removal, including the use of nanoparticles to specifically target cancerous areas [71]. Longer-term goals involve realizing the vision of disease detection at the earliest stage, with devices for testing serum for thousands of cancer markers and precise targeting of cancer cells with specific therapeutics to reduce side effects.

### The Cancer Nanotechnology Plan

Given the interest in applying nanotechnology towards the goal of individualized cancer therapies, the National Cancer Institute (NCI) launched an ambitious initiative in 2004, entitled the Cancer Nanotechnology Plan (CNPlan). Its purpose was to meet the grand challenge goal of eliminating suffering from cancer by 2015, with $144 million in funding allocated for the first five years [72]. While this was a tall order and almost certainly not achievable in the specified timeframe, given the complexities of cancer cells and the paucity of treatments available, this program encapsulated many of the hopes and expectations of nanotechnology. Andrew von Eschenbach, director of the NCI, mentioned that nanotechnology would have "a profound, disruptive effect on how we diagnose, treat, and prevent cancer [72]." To this end, the NCI began to fund multidisciplinary efforts towards improved detection and treatment. The projects initiated under the CNPlan were to be milestone-driven and product oriented, with specific and clear objectives enumerated, to be integrated with developments under the NNI.

In the CNPlan vision statement, the NCI's major stated aim was to "catalyze targeted discovery and development efforts that offer the greatest opportunity for advances in the near and medium terms and to lower the barriers for those advances to be handed off to the private sector for commercial development [72]." The focus of the entire project was business-oriented, with an emphasis on tangible milestones and with the explicit purpose to transfer technologies to industry, in concordance with the post-academic scientific mode. These objectives included the development of imaging agents and diagnostics to detect early stages of cancer and devices for treating localized cancers.

The NCI plan aimed to foster partnerships among academia, industry, and government through the establishment of Centers of Cancer Nanotechnology Excellence (CCNEs) [73]. The purpose of these centers was ostensibly to integrate nanotechnology development into both basic and applied research directed towards clinical cancer treatments with specific milestones. To ensure breadth of research, the NCI required CCNEs to be integrated with a comprehensive cancer center and one or more specialized programs of research excellence (SPORE) and to be affiliated with engineering and physical science research centers [74]. Finally, the NCI expected these centers to foster industry partnerships to facilitate technology transfer from the laboratory to the marketplace. The first set of centers, funded in late 2005, focused mainly on issues of nanoparticle delivery, targeting against specific cancers, and sensitive detection of cancer markers and cells.

The NCI effort represents one of the more ambitious nanotechnology projects funded by the federal government. Although other nations have initiatives for nanobiotechnology, only the United States has specifically designated funding for research into applications towards cancer treatments. As infectious diseases have been largely controlled over the last fifty years, cancer has arisen as one of the major health concerns of the developed world. Concomitantly, due to the complexities of cancer and the relative lack of directed therapeutics, much scientific attention has been focused on understanding the basics. Beginning with the 'War on Cancer' in the early 1970s, the federal government has spent billions of dollars to investigate the pathways of cancer [Note M]. In recent years, there has been a push towards understanding cancer with a systems approach. New instruments such as DNA and protein microarrays have allowed for a global analysis of cancerous cells and a more complete picture of the changes that occur. Likewise, the development of novel therapeutics, including the introduction of monoclonal antibodies against specific tumor markers and factors (such as VEGF or TNF-a) has facilitated specific targeting while reducing systemic toxicity and side effects. These targeted therapeutics were a direct result of an improved molecular understanding of cancer processes.

Because of societal pressures to 'cure cancer,' ample funds are available to address these issues. One result is the expectation that the full weight of science be brought against the complex problems of cancer biology in order to fully treat this vast category of diseases. Even the NCI report succumbed to this pressure and announced its overarching goal as the entire eradication of cancer within ten years. Given the complexities of the disease, and the many forms it can take, this is an unrealistic goal *a priori*, especially within such a constrained timeframe. However, there certainly will be further seminal breakthroughs during this period, and nanotechnology may enable some of these advances.

Despite these advances, will such research investment in the 'nano-war against cancer' ultimately be useful? History is replete with examples of research promoted to solve certain problems, only to find a more complex reality (nuclear energy is one notable example) [Note N]. Many cancer researchers are unaware of nanotechnology's tools, while others are hopeful that these tools will help address certain specific research questions, but are unsure of the more expansive claims of nanobiotechnology's supporters [65,66,69]. Even if useful tools are developed, it will still be many years before they are fully tested and certified for use with patients.

The history of the human genome project may provide some guidance to this concern. With the entire genome now available, it is clear that the sequence information itself means little without other studies to interpret what base pair differences signify in terms of health and disease. Furthermore, most diseases have multi-factorial genetic and environmental causes. The panacea that the initial genome project supporters promised with a complete sequence has not yet been realized; however, this information has proven quite useful to scientific inquiry and has spawned new fields of research, including bioinformatics and computational biology. Though initial results are encouraging, it is impossible to fully predict the impact that nanobiotechnology will have on cancer and in other fields; however, given the trajectory of the HGP, it is clear that the expansion of nanotechnology is far from complete.

## Conclusion

The rise and relative prominence of nanobiotechnology has resulted from a combination of seminal advances in both nanoscience and biotechnology. While the roots of nanotechnology stretch back many years, the explosion of research activity has only occurred within the last fifteen years. Institutionally, the involvement of the federal government through the establishment and subsequent funding of the National Nanotechnology Initiative helped to further the transformation of such research into 'big science.' This model has led to the centralization of equipment and funding resources into large national labs and research centers. As budgetary pressures continue to squeeze science, we will see further consolidation of research. Small groups will continue to exist but will increasingly be crowded by the larger facilities.

We have explored the political and scientific shifts that have enabled the rise of nanotechnology and nanobiotechnology as big science. These financial and epistemic trends have accelerated this transition with a concomitant shift away from the individual researcher towards group-oriented and large-scale science with an increase in contacts between academia and industry. Additionally, federal policy has increasingly emphasized research with economic potential and encouraged technology transfer between universities and commercial firms, providing further incentive for research projects with potential commercial applications. As federal funding becomes increasingly subject to budgetary pressures, industrial support is beginning to represent a more significant fraction of academic funding. The full effect of such a shift remains to be seen, but already we are witnessing a greater emphasis on academic research with practical benefits and commercial potential.

One area in which nanotechnology research has been applied is in the design of technologies for detection and treatment of cancer. Scientists have already begun to create novel nanodevices to detect and treat cancers, though the ultimate impact remains to be seen due to limited exposure in the broader scientific and medical communities. Given the molecular complexities of various cancer types and the amount still unknown, it is rather unlikely that we can completely eliminate cancer in the ten year time frame given by the NCI. Nanotechnology has the potential to impact biological research by allowing scientists an ever-expanding tool set with which to explore the questions of nature, but this outcome is by no means guaranteed.

The scope of nanobiotechnology is continually expanding as scientists develop more applications, especially in the area of nanomedicine and towards the idea of personalized medicine. However, despite the extensive and sometimes fanciful predictions of its boosters, the impact of nanobiotechnology is still extremely limited; we may have to wait many decades some of these promised results, if they even materialize. The example of semiconductor technologies provides an instructive lesson in the time lag between laboratory breakthroughs and widespread use [48]. The first transistor was developed in 1947; however, it took many decades for the transistor and later integrated circuit to be utilized on a massively parallel scale to create computer microchips. It was not until the early 1990s with the explosion of information technology that the greater potential of the transistor technology was realized. Most corporations, university researchers, and government agencies do not have a forty year outlook, yet this is the likely time scale for current nanotechnology development to be fully realized as products if at all. Such implementations will almost undoubtedly be different from how we imagine them now but will be enabled by the discoveries of today.

## Notes

A. In subsequent documents from the NCI, this plan is referred to as the Cancer Nanotechnology Plan (CNPlan). We will refer to this program as the CNPlan henceforth.

B. The terms 'nanoscience' and 'nanotechnology' are often used interchangeably. 'Nanoscience' by itself refers solely to phenomena that occur on the 1–100 nm length scales, which happens to encompass virtually all disciplines of biology as well as many aspects of chemistry and physics. Perhaps the closest 'official' definition of 'nanotechnology' comes from the NNI itself; this version maintains that nanotechnology has three aspects: research and technology development of objects with length scale between 1 and 100 nanometers, creation of devices that have new properties and/or uses due to their small size, and the ability to control such objects on the atomic level. Even this definition is quite expansive in scope, as the length scale encompasses phenomena ranging from single molecular events (at ~1 nm) to bulk materials incorporating thousands of atoms (at ~100 nm), with everything in between also included. In this essay, the terms 'nanoscience' and 'nanotechnology' are used interchangeably.

C. For more detailed information, see the latest updates to the NNI – President's Council of Advisors on Science and Technology: *The National Nanotechnology Initiative at Five Years: Assessment and Recommendations of the National Nanotechnology Advisory Panel*. Washington, D.C.: President's Council of Advisors on Science and Technology, 2005 and National Science and Technology Council,: *The National Nanotechnology Initiative: Strategic Plan*. Washington, D.C.: National Science and Technology Council, 2000.

D. For a detailed description of the processes involved in drafting and passage of the NNI, see McCray.

E. The original texts of these bills are available online at the Library of Congress .

F. The subcommittee's report provides more detail as to the costs and issues relating to the law. See Report of the Committee on Commerce, Science, and Transportation, "21^st ^Century Nanotechnology Research and Development Act," S. 189, (Washington D.C.: Government Printing Office, September 15, 2003).

G. The Center for Information Technology Research in the Interest of Society (CITRIS) was established later as a CalISI.

H. In 1998, the House Committee on Science released a report regarding long-term federal science and technology policy. This report, entitled "Unlocking Our Future," emphasized the priority for federally funded basic research in many areas and the need for improved science education in order to fully leverage America's scientific advantages. In this report, the authors also mentioned that we have now entered the third 'mega-era' of science policy (the first being agricultural research funding before World War II, and the second being science policy during the Cold War), with emphasis on global communication and competitiveness.

I. For an interesting discussion about science policy and its effect on research and development, see Sarewitz D: **Does Science Policy Exist, and if so, Does It Matter? Some Observations on the U.S. R&D Budget. ***Discussion Paper for the Earth Institute Science, Technology, and Global Development Seminar*, April 8, 2003.

J. For more information about this case and others involving scientific freedom and corporate interests, see Krimsky S: *Science in the Private Interest: Has the Lure of Profits Corrupted Biomedical Research? *Lanham, MD: Rowman & Littlefield, 2003. This book is reviewed in Murray TH: **Saving the Soul of Science? ***Nature Biotechnology *2003, **21:**1135–1136.

K. As a result of this episode, the Boots Company ultimately paid over $40 million in damages to states that filed suit to recovery extra money paid for Synthroid when generics would have been as effective. See Wadman M: **$100 m Payout After Drug Data Withheld. ***Nature *1997, **388**: 703.

L. For more information about how passage of the Bayh-Dole act affected university practices regarding patents, see Rai AK, Eisenberg RS: **Bayh-Dole Reform and the Progress of Biomedicine. ***Law and Contemporary Problems *2003, **289**.

M. In 1971, President Nixon declared a war on cancer with passage of the National Cancer Act. This act provided the NCI the authority to carry on a national cancer program and allowed the NCI budget to be approved directly by the president. It also significantly increased funding for cancer-related research.

N. For a good history and background of nuclear energy and its failure to take significant hold in the United States, see Cohn S: *Too Cheap to Meter: An Economic and Philosophical Analysis of the Nuclear Dream*. Albany, NY: State University of New York Press, 1997.
